# Management of pediatric patients admitted for colonic disimpaction: A scoping review protocol

**DOI:** 10.1002/jpr3.12094

**Published:** 2024-06-03

**Authors:** Alaina Berg, Dawn Ebach, Nathaniel A. Justice, Andrew Smelser, Riley Samuelson, Zunaira Mahmood, Aamer Imdad

**Affiliations:** ^1^ University of Iowa Roy J. and Lucille A. Carver College of Medicine Iowa City Iowa USA; ^2^ Department of Pediatrics, Division of Pediatric Gastroenterology, Hepatology, Pancreatology, and Nutrition, Stead Family Children's Hospital University of Iowa Healthcare Iowa City Iowa USA; ^3^ Department of Pediatrics, Division of Pediatric Hospital Medicine, Stead Family Children's Hospital University of Iowa Healthcare Iowa City Iowa USA; ^4^ Department of Pharmaceutical Care University of Iowa Healthcare Iowa City Iowa USA; ^5^ Hardin Library for the Health Sciences University of Iowa Libraries Iowa City Iowa USA

**Keywords:** constipation, enema, laxatives, obstipation, suppositories

## Abstract

**Objectives:**

Chronic constipation is a common condition in pediatric patients worldwide and is associated with decreased quality of life. Inpatient management of constipation is required when outpatient therapy fails and a child becomes obstipated, and unable to pass stool or gas. There is a growing body of evidence regarding different management strategies for pediatric obstipation. This scoping review aims to map the existing literature regarding inpatient management of pediatric obstipation and identify gaps in knowledge.

**Methods:**

We will follow the methodology described by the Joanna Briggs Institute and outlined in the Preferred Reporting Items for Systematic Reviews and Meta‐Analyses extension for Scoping Reviews guidelines. The search strategy will include Embase, PubMed, CINAHL, Cochrane Database of Systematic Reviews, Cochrane Central Register of Controlled Trials, Web of Science, Scopus, and gray literature sources. Two independent reviewers will complete screening for eligible studies in two steps: a scan of the title and abstracts followed by a full‐text review. Studies regarding inpatient management of pediatric obstipation, with experimental or cohort design, and with full text available in English will be included. Systematic reviews will also be included. Two independent reviewers will extract data using a standardized form. Extracted data will be presented in visual and narrative formats, including an evidence map to meet the objectives of this scoping review. This protocol is registered at Open Science Framework.

**Conclusion:**

In this scoping review, we will outline the current evidence available regarding the efficacy and safety of various hospital interventions for the treatment of pediatric obstipation.

## Abstract (highlights)

**What is Known:**

- Constipation is a common pediatric problem and can be treated in the outpatient setting for most patients.
- Inpatient colonic disimpation is done when patients fail outpatient colonic disimpaction.

**What is New:**

- This scoping review will outline current evidence and identify gaps in knowledge regarding inpatient management of pediatric obstipation and will be reported utilizing the Preferred Reporting Items for Systematic Reviews and Meta‐Analyses extension for Scoping Reviews guidelines.
- The search strategy for this review has been developed in collaboration with an experienced medical librarian and includes databases, reference lists, and gray literature sources to ensure all relevant literature is included.

## 1 INTRODUCTION

Functional constipation is a common pediatric problem, diagnosed in 9.5−14.4% of children worldwide based on the Rome IV criteria.^1^, ^2^ Risk factors include low‐fiber diet, low levels of physical activity, exposure to stressful life events, and geographic location, with higher prevalence in North America, South America, and Europe, and lower prevalence in Asia.^1^ It can impact the lives of pediatric patients significantly, with decreased quality of life scores than healthy controls across physical, emotional, social, and school dimensions.^3^, ^4^, ^5^ These patients are also at risk for increased parental stress and decreased family functioning.^6^

Obstipation can occur when chronic constipation has become so severe that impacted hard stool has obstructed the intestines. Treatment aimed at the goal of eliminating hard stool in the distal colon is called disimpaction or a “cleanout.” Accomplishing disimpaction before initiating maintenance laxative therapy has been associated with an increased likelihood of cured constipation.^7^ Current guidelines recommend disimpaction when a patient has encopresis, significant stool mass evident on physical exam or on abdominal radiograph, or a history of incomplete evacuation.^8^

Functional constipation can be managed in the outpatient setting for most patients with education, dietary modifications, behavioral modifications, and oral pharmacotherapy.^9^, ^10^ When patients fail outpatient management of chronic constipation and impaction of hard stool occurs, hospitalization is required.^10^, ^11^ Risk factors for failing outpatient treatment of constipation and requiring hospitalization include Black ethnicity, prematurity, developmental delay, and the presence of overflow incontinence.^10^ The rates of hospitalization for constipation have been increasing in the pediatric population: the frequency of admission for constipation in 1997 was 17 per 10,000 patients but increased to 51 per 10,000 patients by 2010.^12^ Benefits of inpatient treatment include the ability to complete more invasive procedures, such as the placement of a nasogastric tube to facilitate the delivery of a substantial volume of osmotic laxative solution,^11^ the delivery of intravenous fluids, athe administration of rectal therapies including enemas and manual disimpaction. However, inpatient treatment is expensive,^12^ and its treatments are often uncomfortable for the patient; it should only be utilized when required.

There is a growing number of studies and reviews of studies that compare different inpatient disimpaction strategies, including the use of various osmotic and/or stimulant laxatives,^11^, ^13^, ^14^, ^15^, ^16^, ^17^ the addition of rectal therapies,^17^, ^18^, ^19^, ^20^, ^21^, ^22^ and the use of supportive care (e.g., intravenous fluids).^11^, ^13^

We aim to map the available evidence on different interventions used for inpatient management of pediatric obstipation and identify gaps in current knowledge.

## 2 METHODS

This protocol was developed a priori using the Joanna Briggs Institute Methodology^23^ and reported using the Preferred Reporting Items for Systematic Reviews and Meta‐Analyses extension for Scoping Reviews (PRISMA‐ScR) guidelines.^24^ The protocol was reviewed by members of the research team and registered prospectively with Open Science Framework on February 15, 2024 (registrationhttps://doi.org/10.17605/OSF.IO/KEQV5).

## 3 RESEARCH QUESTION

The objective of this review is to outline the available evidence and identify current gaps in knowledge regarding inpatient management of pediatric obstipation. To accomplish this objective, we developed the following research questions:

1. How do pharmacologic interventions, delivered via the upper gastrointestinal tract, compare in effectiveness in pediatric patients with obstipation admitted for inpatient cleanout?
2. How do pharmacologic interventions, delivered via the rectum, compare in effectiveness in pediatric patients with obstipation admitted for inpatient cleanout?
3. How do supportive care interventions benefit pediatric patients with obstipation admitted for inpatient cleanout?

**Figure 1:**
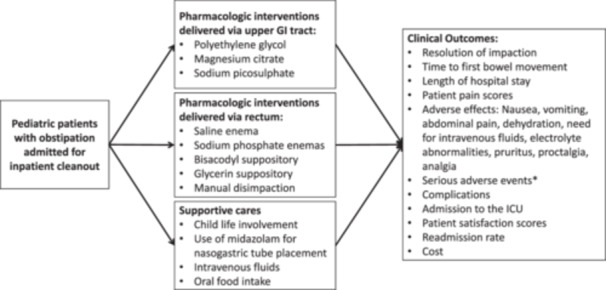
Conceptual framework for scoping review. *Serious adverse events are defined by FDA guidelines and include death or life‐threatening events or an event leading to long‐term disability.^25^ FDA, Food and Drug Administration; GI, gastrointestinal; ICU, intensive care unit.

The following research questions are also represented in a conceptual framework (Figure 1).

## 4 ELIGIBILITY CRITERIA

### 4.1 Type of population

Peer‐reviewed articles that focus on pediatric patients with chronic constipation or obstipation (defined as severe constipation with inability to pass stool or gas) treated in the inpatient setting will be included. Studies that do not include pediatric patients (defined as ≤18 years old) will be excluded. Studies that describe only chronic management of constipation without reference to obstipation or inpatient disimpaction will be excluded.

### 4.2 Type of intervention and comparator

Studies involving any disimpaction intervention including but not limited to polyethylene glycol solution, magnesium citrate solution, sodium picosulphate stimulant laxative, sodium phosphate enemas, saline enemas, bisacodyl suppositories, glycerin suppositories, or manual disimpaction, and supportive cares of intravenous fluids, oral food intake, child life involvement, and/or the use of midazolam for nasogastric tube placement in comparison to each other, placebo, no intervention or standard of care. Surgical interventions will not be included.

### 4.3 Types of studies

Experimental study designs (randomized and quasi‐experimental), cohort studies, systematic reviews, and hospital protocols will be eligible for study. Case‐control studies, opinion pieces, and conference abstracts will also be excluded. Additionally, articles must have full text available in English. No publication date limit will be in place for studies to be included in this review.

### 4.4 Types of outcomes measures

Articles must report any of the following outcomes to be included in the study: resolution of impaction, time to first bowel movement, length of hospital stay, patient pain scores, adverse effects (nausea, vomiting, abdominal pain, dehydration, need for intravenous fluids, electrolyte abnormalities, pruritis, proctalgia, analgia), serious adverse events,^25^ complications, admission to the intensive care unit, patient satisfaction scores, readmission rate, or cost.

### 4.5 Information sources

To identify relevant articles, the following databases will be searched: Embase, PubMed, CINAHL, Cochrane Database of Systematic Reviews, Cochrane Central Register of Controlled Trials, Web of Science, and Scopus. An experienced medical information specialist drafted the search strategy. Research team members further refined the strategy. A smell set of preselected articles meeting inclusion criteria will be used to validate the searches. The final search strategy is included with this protocol (See Document, Supplemental Digital Content 1, which includes the search queries used for each database).

### 4.6 Other sources

Additional articles will be identified by scanning relevant review articles, and gray literature will be searched for using clinicaltrials.gov and Google Scholar to supplement the search and identify all relevant evidence. We will also search for inpatient cleanout protocols using a Google search and searching the websites of the top 10 children's hospitals in the United States as defined by the US World News 2023−2024 report.^26^ All identified articles will be exported to EndNote and duplicates will be removed.

## 5 STUDY RECORDS

### 5.1 Study selection process

A two‐step process of screening will complete the selection of eligible studies. First, two independent reviewers will screen the title and abstracts of all articles to identify those relevant to this scoping review according to the eligibility criteria described above. Studies that meet inclusion criteria or can not be excluded based on title and abstract will be further evaluated. When there is insufficient published data available to determine if a study should be included or excluded, we will contact authors via email to solicit additional data and ask permission to report any data shared with us.

After the initial screening of the titles and abstracts, two independent reviewers will assess the full text of the remaining sources and determine inclusion eligibility. A third reviewer will be involved in discussing and resolving any disagreements between the two independent reviewers during the selection of relevant sources. For each full text that is excluded, a reason for exclusion will be recorded (e.g., wrong setting, wrong patient population, etc.).

We will present the number of studies excluded at each step of the selection process and the reason for the exclusion of full texts in a flow diagram according to the PRISMA‐ScR guidelines.^24^ This flow diagram will also include information about how many authors were contacted, how many responded, and how many authors sent data.

### 5.2 Data collection and management process

Relevant data will be extracted from eligible studies and charted on a data charting form by two independent reviewers. The data charting form will be developed by the reviewers. The two independent reviewers will pilot the data extraction form using two studies. Then a third independent extractor will meet with the two extractors to discuss, resolve any conflicts, and revise the data extraction form as needed. Both independent extractors will then complete full data extraction.

Reviewers will contact authors as needed throughout data extraction to solicit additional information if published information is insufficient for the review. The two reviewers will compare and discuss their final extraction results and any disagreements that can not be resolved by discussion will be resolved by a third reviewer. The final extracted data will be shared with all members of the research team. The extracted data will be attached to the final report in an XLSX file.

## 6 DATA ITEMS

Two independent reviewers will extract data utilizing a form that will include article characteristics (e.g., title, first author name, year published, type of study, single or multi‐center, country of study, year of study, sample size, intervention and control dose, frequency, route of administration) and clinical outcomes (e.g., frequency of bowel movements, duration of hospital stay, length of treatment, adverse effects, complications, need for intravenous access, patient satisfaction scores). The final report will include a “Characteristics of Included Studies” table.

## 7 DATA SYNTHESIS

After data extraction is complete, articles will be organized by intervention and study design and presented in a table with an accompanying narrative summary. Evidence mapping is a useful tool for visualizing current research and identifying areas in need of further investigation.^27^ We will utilize the EPPI‐Mapper tool to create a map of the evidence.^28^ We will report the certainty of evidence via the Grades of Recommendation, Assessment, Development, and Evaluation (GRADE) guidelines^29^ for studies that have been evaluated by a systematic review. For those studies not already evaluated for risk of bias in a systematic review, we will complete this with the GRADE evaluation.^29^ All findings will be reported in line with the PRISMA‐ScR checklist.^24^

## 8 DISSEMINATION

We plan to disseminate research findings via peer‐reviewed publications and presentations at relevant conferences.

## 9 CONCLUSION

This scoping review will outline current available evidence regarding the inpatient management of pediatric obstipation. This information will direct future research efforts toward areas with gaps in research as well as guide clinicians who treat children with obstipation.

## CONFLICT OF INTEREST STATEMENT

The authors declare no conflict of interest.

## ETHICS STATEMENT

Ethics approval is not required for this scoping review as no primary data will be collected. No patient consent for publication is required.

## Supporting information


**Document, Supplementary Digital Content 1**: Search queries for each database.
