# Supplementary material for: Management of pediatric patients admitted for colonic disimpaction: A scoping review protocol
Source: JPGN Rep. 2024 Jun 3;5(3):265–9. doi: 10.1002/jpr3.12094 (PMC11322030; doi:10.1002/jpr3.12094)
Supplement: Supplementary file 1 — Document, Supplementary Digital Content 1: Search queries for each database. [file JPR3-5-265-s001.docx]

PUBMED:

("Constipation"[Mesh] OR “constipat*”[tiab] OR “obstipat*”[tiab] OR “Colonic Inertia”[tiab] OR “hard feces”[tiab] OR “hard faeces”[tiab] OR “hard stool*”[tiab] OR “impacted stool*”[tiab] OR “impacted feces”[tiab] OR “impacted faeces”[tiab] OR “feces impact*”[tiab] OR “faeces impact*”[tiab] OR "Fecal Impaction"[Mesh] OR “fecal impact*”[tiab] OR “faecal impact*”[tiab] OR “obstructed defec*”[tiab] OR “obstructed colon*”[tiab] OR “obstructed bowel*”[tiab] OR “obstructed outlet*”[tiab] OR “colon obstruction*”[tiab] OR “colonic obstruction*”[tiab] OR “bowel obstruction*”[tiab] OR “outlet obstruction*”[tiab] OR “disordered defec*”[tiab] OR “disordered defaec*”[tiab] OR “coprostasis”[tiab] OR "Encopresis"[Mesh] OR "Encopresis"[tiab] OR “faeces incontin*”[tiab] OR “feces incontin*”[tiab] OR “fecal incontin*”[tiab] OR “faecal incontin*”[tiab] OR “anal incontin*”[tiab] OR “bowel incontin*”[tiab] OR “incontinentia alvi”[tiab])

AND

("Constipation/drug therapy"[Mesh] OR "Constipation/therapy"[Mesh] OR “disimpact*”[tiab] OR "constipation management"[tiab] OR "constipation therap*"[tiab] OR "Laxatives"[Mesh] OR “laxa*”[tiab] OR “lavage*”[tiab] OR “irrigat*”[tiab] OR “intestinal clear*”[tiab] OR “evacuant*”[tiab] OR "Cathartics"[Mesh] OR “cathartic*”[tiab] OR “purgative*”[tiab] OR "Enema"[Mesh] OR “enema*”[tiab] OR “Bowel Preparation Solution*”[tiab] OR "Suppositories"[Mesh] OR “suppositor*”[tiab] OR “cleanout*”[tiab] OR “clean out*”[tiab] OR “cleaning”[tiab] OR "Administration, Intravenous"[Mesh] OR “intravenous”[tiab] OR “Drip Infusion*”[tiab] OR "Administration, Rectal"[Mesh] OR “rectal administration”[tiab] OR “anal administration”[tiab] OR “rectal drug*”[tiab] OR “rectum drug*”[tiab] OR “anal drug*”[tiab] OR “anorectal drug*”[tiab] OR “rectal Instillation*”[tiab] OR “anal Instillation*”[tiab] OR “anorectal Instillation*”[tiab] OR “rectal therap*”[tiab] OR “per rectum”[tiab] OR “by rectum”[tiab] OR “child life”[tiab] OR “digestive tract intubation”[tiab] OR “digestive tract tube*”[tiab] OR "Intubation, Gastrointestinal"[Mesh] OR “gastrointestinal intubation”[tiab] OR “gastrointestinal tube*”[tiab] OR “nasogastric*”[tiab] OR “naso gastric*”[tiab] OR “nasoenter*”[tiab] OR “naso enter*”[tiab] OR “NGT”[tiab] OR “NG tube”[tiab] OR “nasojejunal”[tiab] OR “naso jejunal”[tiab] OR “NJT”[tiab] OR “Flexiflo”[tiab] OR “orogastric*”[tiab] OR “oroenteric*”[tiab] OR “intestinal decompression”[tiab] OR “intestine decompression”[tiab] OR “colon decompression”[tiab] OR “colonic decompression”[tiab])

AND

("Pediatrics"[Mesh] OR “pediat*”[tiab] OR “paediat*”[tiab] OR "Hospitals, Pediatric"[Mesh] OR "Child"[Mesh] OR “child*”[tiab] OR “Juvenile*”[tiab] OR “toddler*”[tiab] OR "Adolescent"[Mesh] OR “Adolescen*”[tiab] OR “Teen*”[tiab])

EMBASE:

('constipation'/exp OR ‘constipat*’:ab,ti,kw OR ‘obstipat*’:ab,ti,kw OR ‘Colonic Inertia’:ab,ti,kw OR 'hard feces'/exp OR ‘hard feces’:ab,ti,kw OR ‘hard faeces’:ab,ti,kw OR ‘hard stool*’:ab,ti,kw OR 'feces impaction'/exp OR ‘impacted stool*’:ab,ti,kw OR ‘impacted feces’:ab,ti,kw OR ‘impacted faeces’:ab,ti,kw OR ‘feces impact*’:ab,ti,kw OR ‘faeces impact*’:ab,ti,kw OR ‘fecal impact*’:ab,ti,kw OR ‘faecal impact*’:ab,ti,kw OR ‘obstructed defec*’:ab,ti,kw OR ‘obstructed colon*’:ab,ti,kw OR ‘obstructed bowel*’:ab,ti,kw OR ‘obstructed outlet*’:ab,ti,kw OR 'colon obstruction'/exp OR ‘colon obstruction*’:ab,ti,kw OR ‘colonic obstruction*’:ab,ti,kw OR ‘bowel obstruction*’:ab,ti,kw OR ‘outlet obstruction*’:ab,ti,kw OR ‘disordered defec*’:ab,ti,kw OR ‘disordered defaec*’:ab,ti,kw OR ‘coprostasis’:ab,ti,kw OR 'feces incontinence'/exp OR ‘Encopresis’:ab,ti,kw OR ‘feces incontin*’:ab,ti,kw OR ‘faeces incontin*’:ab,ti,kw OR ‘fecal incontin*’:ab,ti,kw OR ‘faecal incontin*’:ab,ti,kw OR ‘anal incontin*’:ab,ti,kw OR ‘bowel incontin*’:ab,ti,kw OR ‘incontinentia alvi’:ab,ti,kw)

AND

('constipation therapy'/exp OR ‘disimpact*’:ab,ti,kw OR ‘constipation management’:ab,ti,kw OR ‘constipation therap*’:ab,ti,kw OR 'laxative'/exp OR ‘laxa*’:ab,ti,kw OR 'lavage'/exp OR ‘lavage*’:ab,ti,kw OR ‘irrigat*’:ab,ti,kw OR ‘intestinal clear*’:ab,ti,kw OR ‘evacuant*’:ab,ti,kw OR ‘cathartic*’:ab,ti,kw OR ‘purgative*’:ab,ti,kw OR 'enema'/exp OR ‘enema*’:ab,ti,kw OR ‘Bowel Preparation Solution*’:ab,ti,kw OR 'suppository'/exp OR ‘suppositor*’:ab,ti,kw OR ‘cleanout*’:ab,ti,kw OR ‘clean out*’:ab,ti,kw OR ‘cleaning’:ab,ti,kw OR 'intravenous drug administration'/exp OR ‘intravenous’:ab,ti,kw OR ‘Drip Infusion*’:ab,ti,kw OR 'rectal drug administration'/exp OR ‘rectal administration’:ab,ti,kw OR ‘anal administration’:ab,ti,kw OR ‘rectal drug*’:ab,ti,kw OR ‘rectum drug*’:ab,ti,kw OR ‘anal drug*’:ab,ti,kw OR ‘anorectal drug*’:ab,ti,kw OR ‘rectal Instillation*’:ab,ti,kw OR ‘anal Instillation*’:ab,ti,kw OR ‘anorectal Instillation*’:ab,ti,kw OR ‘rectal therap*’:ab,ti,kw OR ‘per rectum’:ab,ti,kw OR ‘by rectum’:ab,ti,kw OR 'child life specialist'/exp OR ‘child life’:ab,ti,kw OR 'digestive tract intubation'/exp OR 'digestive tract intubation':ab,ti,kw OR 'digestive tract tube*':ab,ti,kw OR 'gastrointestinal intubation tube'/exp OR 'gastrointestinal intubation’:ab,ti,kw OR 'gastrointestinal tube*’:ab,ti,kw OR ‘nasogastric*’:ab,ti,kw OR ‘naso gastric*’:ab,ti,kw OR ‘nasoenter*’:ab,ti,kw OR ‘naso enter*’:ab,ti,kw OR ‘NGT’:ab,ti,kw OR ‘NG tube’:ab,ti,kw OR ‘nasojejunal’:ab,ti,kw OR ‘naso jejunal’:ab,ti,kw OR ‘NJT’:ab,ti,kw OR ‘Flexiflo’:ab,ti,kw OR ‘orogastric*’:ab,ti,kw OR ‘oroenteric*’:ab,ti,kw OR 'intestinal decompression':ab,ti,kw OR 'intestine decompression':ab,ti,kw OR 'colon decompression':ab,ti,kw OR 'colonic decompression':ab,ti,kw)

AND

('pediatrics'/exp OR ‘pediat*’:ab,ti,kw OR ‘paediat*’:ab,ti,kw OR 'child'/exp OR ‘child*’:ab,ti,kw OR 'juvenile'/exp OR ‘Juvenile*’:ab,ti,kw OR ‘toddler*’:ab,ti,kw OR 'adolescent'/exp OR ‘Adolescen*’:ab,ti,kw OR ‘Teen*’:ab,ti,kw)

SCOPUS:

(

(

INDEXTERMS(“constipation” OR “hard feces” OR “feces impaction” OR “colon obstruction” OR “feces incontinence”) OR TITLE-ABS-KEY( “constipat*” OR “obstipat*” OR “Colonic Inertia” OR “hard feces” OR “hard faeces” OR “hard stool*” OR “impacted stool*” OR “impacted feces” OR “impacted faeces” OR “feces impact*” OR “faeces impact*” OR “fecal impact*” OR “faecal impact*” OR “obstructed defec*” OR “obstructed colon*” OR “obstructed bowel*” OR “obstructed outlet*” OR “colon obstruction*” OR “colonic obstruction*” OR “bowel obstruction*” OR “outlet obstruction*” OR “disordered defec*” OR “disordered defaec*” OR “coprostasis” OR “Encopresis” OR “feces incontin*” OR “faeces incontin*” OR “fecal incontin*” OR “faecal incontin*” OR “anal incontin*” OR “bowel incontin*” OR “incontinentia alvi”)

)

AND

(

INDEXTERMS(“constipation therapy” OR “laxative” OR “lavage” OR “enema” OR “suppository” OR “intravenous drug administration” OR “rectal drug administration” OR “child life specialist” OR “digestive tract intubation” OR “gastrointestinal intubation tube”) OR TITLE-ABS-KEY(“disimpact*” OR “constipation management” OR “constipation therap*” OR “laxa*” OR “lavage*” OR “irrigat*” OR “intestinal clear*” OR “evacuant*” OR “cathartic*” OR “purgative*” OR “enema*” OR “Bowel Preparation Solution*” OR “suppositor*” OR “cleanout*” OR “clean out*” OR “cleaning” OR “intravenous” OR “Drip Infusion*” OR “rectal administration” OR “anal administration” OR “rectal drug*” OR “rectum drug*” OR “anal drug*” OR “anorectal drug*” OR “rectal Instillation*” OR “anal Instillation*” OR “anorectal Instillation*” OR “rectal therap*” OR “per rectum” OR “by rectum” OR “child life” OR “digestive tract intubation” OR “digestive tract tube*” OR “gastrointestinal intubation” OR “gastrointestinal tube*” OR “nasogastric*” OR “naso gastric*” OR “nasoenter*” OR “naso enter*” OR “NGT” OR “NG tube” OR “nasojejunal” OR “naso jejunal” OR “NJT” OR “Flexiflo” OR “orogastric*” OR “oroenteric*” OR “intestinal decompression” OR “intestine decompression” OR “colon decompression” OR “colonic decompression”)

)

AND

(

INDEXTERMS(“pediatrics” OR “child” OR “juvenile” OR “adolescent”) OR TITLE-ABS-KEY( “pediat*” OR “paediat*” OR “child*” OR “Juvenile*” OR “toddler*” OR “Adolescen*” OR “Teen*”)

)

)

CINAHL:

((MH "Constipation+") OR (“constipat*”) OR (“obstipat*”) OR (“Colonic Inertia”) OR (“hard feces”) OR (“hard faeces”) OR (“hard stool*”) OR (MH "Feces, Impacted") OR (“impacted stool*”) OR (“impacted feces”) OR (“impacted faeces”) OR (“feces impact*”) OR (“faeces impact*”) OR (“fecal impact*”) OR (“faecal impact*”) OR (“obstructed defec*”) OR (“obstructed colon*”) OR (“obstructed bowel*”) OR (“obstructed outlet*”) OR (“colon obstruction*”) OR (“colonic obstruction*”) OR (“bowel obstruction*”) OR (“outlet obstruction*”) OR (“disordered defec*”) OR (“disordered defaec*”) OR (“coprostasis”) OR (MH "Fecal Incontinence") OR ("Encopresis”) OR (“faeces incontin*”) OR (“feces incontin*”) OR (“fecal incontin*”) OR (“faecal incontin*”) OR (“anal incontin*”) OR (“bowel incontin*”) OR (“incontinentia alvi”))

AND

((MH "Fecal Impaction Removal") OR (“disimpact*”) OR (“constipation management”) OR (“constipation therap*”) OR (MH "Cathartics+") OR (“laxa*”) OR (“lavage*”) OR (“irrigat*”) OR (“intestinal clear*”) OR (“evacuant*”) OR (“cathartic*”) OR (“purgative*”) OR (MH "Enema") OR (“enema*”) OR (“Bowel Preparation Solution*”) OR (MH "Suppositories") OR (“suppositor*”) OR (“cleanout*”) OR (“clean out*”) OR (“cleaning”) OR (MH "Administration, Intravenous") OR (“intravenous”) OR (“Drip Infusion*”) OR (MH "Administration, Rectal") OR (“rectal administration”) OR (“anal administration”) OR (“rectal drug*”) OR (“rectum drug*”) OR (“anal drug*”) OR (“anorectal drug*”) OR (“rectal Instillation*”) OR (“anal Instillation*”) OR (“anorectal Instillation*”) OR (“rectal therap*”) OR (“per rectum”) OR (“by rectum”) OR (“child life”) OR (MH "Intubation, Gastrointestinal") OR (“digestive tract intubation”) OR (“digestive tract tube*”) OR (“gastrointestinal intubation”) OR (“gastrointestinal tube*”) OR (MH "Nasoenteral Tubes") OR (“nasogastric*”) OR (“naso gastric*”) OR (“nasoenter*”) OR (“naso enter*”) OR (“NGT”) OR (“NG tube”) OR (“nasojejunal”) OR (“naso jejunal”) OR (“NJT”) OR (“Flexiflo”) OR (“orogastric*”) OR (“oroenteric*”) OR (“intestinal decompression”) OR (“intestine decompression”) OR (“colon decompression”) OR (“colonic decompression”))

AND

((MH "Pediatrics+") OR (“pediat*”) OR (“paediat*”) OR (MH "Hospitals, Pediatric") OR (MH "Child+") OR (“child*”) OR (“Juvenile*”) OR (“toddler*”) OR (MH "Adolescence+") OR (“Adolescen*”) OR (“Teen*”))

COCHRANE:

Title Abstract Keyword searches:

(

constipat* OR obstipat* OR “Colonic Inertia” OR “hard feces” OR “hard faeces” OR (hard NEXT stool*) OR (impacted NEXT stool*) OR “impacted feces” OR “impacted faeces” OR (feces NEXT impact*) OR (faeces NEXT impact*) OR (fecal NEXT impact*) OR (faecal NEXT impact*) OR (obstructed NEXT defec*) OR (obstructed NEXT colon*) OR (obstructed NEXT bowel*) OR (obstructed NEXT outlet*) OR (colon NEXT obstruction*) OR (colonic NEXT obstruction*) OR (bowel NEXT obstruction*) OR (outlet NEXT obstruction*) OR (disordered NEXT defec*) OR (disordered NEXT defaec*) OR coprostasis OR Encopresis OR (faeces NEXT incontin*) OR (feces NEXT incontin*) OR (fecal NEXT incontin*) OR (faecal NEXT incontin*) OR (anal NEXT incontin*) OR (bowel NEXT incontin*) OR “incontinentia alvi”

)

AND

(

disimpact* OR "constipation management" OR (constipation NEXT therap*) OR laxa* OR lavage* OR irrigat* OR (intestinal NEXT clear*) OR evacuant* OR cathartic* OR purgative* OR enema* OR (“Bowel Preparation” NEXT Solution*) OR suppositor* OR cleanout* OR (clean NEXT out*) OR cleaning OR intravenous OR (Drip NEXT Infusion*) OR “rectal administration” OR “anal administration” OR (rectal NEXT drug*) OR (rectum NEXT drug*) OR (anal NEXT drug*) OR (anorectal NEXT drug*) OR (rectal NEXT Instillation*) OR (anal NEXT Instillation*) OR (anorectal NEXT Instillation*) OR (rectal NEXT therap*) OR “per rectum” OR “by rectum” OR “child life” OR “digestive tract intubation” OR (“digestive tract” NEXT tube*) OR “gastrointestinal intubation” OR (gastrointestinal NEXT tube*) OR nasogastric* OR (naso NEXT gastric*) OR nasoenter* OR (naso NEXT enter*) OR NGT OR “NG tube” OR nasojejunal OR “naso jejunal” OR NJT OR “Flexiflo” OR orogastric* OR oroenteric* OR “intestinal decompression” OR “intestine decompression” OR “colon decompression” OR “colonic decompression”

)

AND

(

pediat* OR paediat* OR child* OR Juvenile* OR toddler* OR Adolescen* OR Teen*

)

WEB OF SCIENCE:

Topic searches:

(“constipat*” OR “obstipat*” OR “Colonic Inertia” OR “hard feces” OR “hard faeces” OR “hard stool*” OR “impacted stool*” OR “impacted feces” OR “impacted faeces” OR “feces impact*” OR “faeces impact*” OR “fecal impact*” OR “faecal impact*” OR “obstructed defec*” OR “obstructed colon*” OR “obstructed bowel*” OR “obstructed outlet*” OR “colon obstruction*” OR “colonic obstruction*” OR “bowel obstruction*” OR “outlet obstruction*” OR “disordered defec*” OR “disordered defaec*” OR “coprostasis” OR "Encopresis" OR “faeces incontin*” OR “feces incontin*” OR “fecal incontin*” OR “faecal incontin*” OR “anal incontin*” OR “bowel incontin*” OR “incontinentia alvi”)

AND

(“disimpact*” OR "constipation management" OR "constipation therap*" OR “laxa*” OR “lavage*” OR “irrigat*” OR “intestinal clear*” OR “evacuant*” OR “cathartic*” OR “purgative*” OR “enema*” OR “Bowel Preparation Solution*” OR “suppositor*” OR “cleanout*” OR “clean out*” OR “cleaning” OR “intravenous” OR “Drip Infusion*” OR “rectal administration” OR “anal administration” OR “rectal drug*” OR “rectum drug*” OR “anal drug*” OR “anorectal drug*” OR “rectal Instillation*” OR “anal Instillation*” OR “anorectal Instillation*” OR “rectal therap*” OR “per rectum” OR “by rectum” OR “child life” OR “digestive tract intubation” OR “digestive tract tube*” OR “gastrointestinal intubation” OR “gastrointestinal tube*” OR “nasogastric*” OR “naso gastric*” OR “nasoenter*” OR “naso enter*” OR “NGT” OR “NG tube” OR “nasojejunal” OR “naso jejunal” OR “NJT” OR “Flexiflo” OR “orogastric*” OR “oroenteric*” OR “intestinal decompression” OR “intestine decompression” OR “colon decompression” OR “colonic decompression”)

AND

(“pediat*” OR “paediat*” OR “child*” OR “Juvenile*” OR “toddler*” OR “Adolescen*” OR “Teen*”)
